# Suicide in the Philippines: time trend analysis (1974-2005) and literature review

**DOI:** 10.1186/1471-2458-11-536

**Published:** 2011-07-06

**Authors:** Maria Theresa Redaniel, May Antonnette Lebanan-Dalida, David Gunnell

**Affiliations:** 1 School of Social and Community Medicine, University of Bristol, Canynge Hall, 39 Whatley Road, Bristol, BS8 2PS, UK; 2 Department of Epidemiology and Biostatistics, University of the Philippines-Manila, 625 Pedro Gil St, Ermita, Manila, 1000, Philippines

## Abstract

**Background:**

Suicide prevention is given a low priority in many Western Pacific countries due to competing health problems, stigma and poor understanding of its incidence and aetiology. Little is known about the epidemiology of suicide and suicidal behaviour in the Philippines and although its incidence is reported to be low, there is likely to be under-reporting because of its non-acceptance by the Catholic Church and the associated stigma to the family. This study aims to investigate trends in the incidence of suicide in the Philippines, assess possible underreporting and provide information on the methods used and the reasons for suicide.

**Methods:**

Data for suicide deaths occurring between 1974 and 2005 were obtained from Philippine Health Statistics. Age- and sex-specific trends were examined graphically. Underreporting was investigated by comparing trends in suicides, accidents and deaths of undetermined intent. To provide a fuller picture of suicide in the Philippines, a comprehensive search for published papers, theses and reports on the epidemiology of suicide in the Philippines was undertaken.

**Results:**

The incidence of suicide in males increased from 0.23 to 3.59 per 100,000 between 1984 and 2005. Similarly, rates rose from 0.12 to 1.09 per 100,000 in females. Amongst females, suicide rates were highest in 15-24 year olds, whilst in males rates were similar in all age groups throughout the study period. The most commonly used methods of suicide were hanging, shooting and organophosphate ingestion. In non-fatal attempts, the most common methods used were ingestion of drugs, specifically isoniazid and paracetamol, or organophosphate ingestion. Family and relationship problems were the most common precipitants. While rates were lower compared to other countries, there is suggestive evidence of underreporting and misclassification to undetermined injury. Recent increases may reflect either true increase or better reporting of suicides.

**Conclusions:**

While suicide rates are low in the Philippines, increases in incidence and relatively high rates in adolescents and young adults point to the importance of focused suicide prevention programs. Improving data quality and better reporting of suicide deaths is likewise imperative to inform and evaluate prevention strategies.

## Background

Suicide is a major contributor to premature mortality worldwide and is among the leading causes of death in the Western Pacific Region [1]. Approximately 32% of the world's suicides occur in the region, and its annual incidence of 19.3 per 100,000 is 30% higher than the global average [2]. While acknowledged as an important and neglected health issue, it remains a low priority in most Western Pacific countries due to competing health problems, stigma and poor understanding of the condition [3].

The Philippines, with a population of approximately 90 million, is one of the most populous countries in the Western Pacific, yet very little is known about the epidemiology of suicide and suicidal behaviour in the country [4]. The only predominantly Catholic country in Asia, it is an archipelago of 7,106 islands, with 66% of the population living in urban areas [4-6]. Around 33% of the population are impoverished, in spite of reported economic growth in recent years [5].

Official suicide rates are lower in the Philippines than in many other countries in the Western Pacific region [7], although there is likely to be under-reporting because of its non-acceptance by the Catholic church and the associated disgrace and stigma to the family [8]. As in other Catholic countries, a high proportion of suicide deaths are likely to be misclassified as injury of undetermined intent or accidents [9]. A systematic analysis of the possible underreporting of suicides is important so its true incidence and trends can be estimated. To date, no studies of national trends in the incidence of suicide or the national epidemiology of suicidal behaviour have been undertaken using Philippine mortality data. Such an analysis is important both to provide a more complete picture of the size of the problem and to facilitate better informed decisions concerning priorities for prevention such as high risk age/sex groups and popular suicide methods that are potentially amenable to method-restriction policies.

## Methods

### Data sources

Data on deaths from suicide, accidental poisoning, other accidents, and injury of undetermined intent occurring between 1974-2005 were obtained from the Philippine Health Statistics (PHS) produced by the Department of Health (DOH) [10]. Data for the Health Statistics were provided by the National Statistics Office (NSO), which is mandated by the Civil Registry Law (Republic Act No. 3753) to register all vital events in the country [11-13].

In the Philippines, all deaths must be certified by the physician who last attended the deceased [13]. For deaths occurring outside hospital, certification by the health officer is based on the symptoms prior to death and circumstances of the death as reported by the relatives or friends of the deceased [12]. Each vital event is registered in the Local Civil Registrar Offices (LCRO) [13], each serving a population of 25,000 for municipalities and 150,000 for cities. The LCRO sends a copy of each death certificate to the Office of the Civil Registrar General of the NSO for processing and archiving [11,13]. The NSO provides the DOH with summary tables of the numbers of deaths and population estimates by cause, age and sex [11].

While data are available for earlier years, it is only after 1974 that the collection of mortality statistics was centralized to the National Statistics Office [12]. This year was therefore chosen as the starting year for the analysis. Codes used by the National Statistics Office to classify suicides and injuries follow the International Classification of Diseases (ICD) and are shown in Table 1.

**Table 1:** Codes used for the classification of cause of death as suicide, accidental poisoning, other accidents and undetermined injury.

Cause	ICD 8 (1974-1986)	ICD 9 (1987-1996)	ICD 10 (1997-2005)
Suicide	E950-E959	E950-E959	X60-X84
Accidental poisoning	E850-E877	E850-E869	X40-X49
Other accidents	E900-E909; E911-E918; E929-E949	E870-E876; E878-E879; E916-E949	W20-W49; Y40-Y89 (1997-1998) W20-W64; W75-W99; X10-X29; X50-X59; X85-Y86 (1999-2005)
Injury undetermined	E980-E989	E980-E989	Y10-Y34

Age-standardized rates were computed using the WHO world standard population [14]. To smooth the annual rates, three year moving averages, centred on the last year of the each 3-year period, were computed, so all graphs begin in 1976. Age-specific rates for five-year periods were also computed.

Possible underreporting was investigated by comparing trends in suicides with deaths due to accidents and deaths of undetermined intent. Similarities in trends across different cause of death categories or increases in the incidence of one cause of death accompanied by a reciprocal decline in another were used as indirect evidence of under-reporting.

### Literature review

A comprehensive search for published papers on suicide in the Philippines was carried out using Medline and local online research databases (the Philippine e-Library, the DOH e-Library and HERDIN) for the years 1960 to 2010. The following terms were used for the database searches: suicide** (covers suicidal), self (covers self-harm/self-poisoning), overdose, poison, poisoning, pesticide*, insecticide*, rodenticide*, paraquat, organophosphate*, organophosphorus*, agrochemical*. As pesticide self-poisoning and overdose of medicines are reported as the most commonly used suicide methods in the Western Pacific Region [3], poisoning terms were added in the keywords. This search was supplemented by searches using the Philippine Index Medicus, the WHO catalogues, databases of the University of the Philippines Manila and Diliman and the Philippine Information Agency (PIA) and Google. Additional papers were identified from the list of references of the obtained articles, monographs and theses. Papers were in English as this is the language used for scientific publications in the Philippines.

A total of 92 papers, theses and monographs were identified through the searches. Of these, 20 were not relevant, 4 could not be traced and 68 were retrieved. Only one paper on suicide deaths was identified - an anthropological study in an indigenous population [15]. Only four studies focused on attempted suicide, one from an urban area (n = 113) and three from areas transitioning from peri-urban to urban at the time of the study (n = 55-130) [16-19].

To supplement the limited information on suicides available from national statistics (e.g. no information on method of suicide), newspaper reports of suicides were reviewed. This approach has been used previously in countries where suicide is stigmatized and underreported, and where sources of suicide information are limited [20]. In the Philippines, news reports may provide information on the suicide such as the method used - this is not reported in the national mortality statistics. These articles were written by reporters covering police stations and were based on police reports. Using the Philippine e-library database, magazine and newspaper articles (mostly from nationally circulated publications) on suicides and self-harm from 1986 to 2010 were searched. A total of 122 were identified, 6 could not be traced and 116 were retrieved.

## Results

### Suicide Rates and Trends

**Figure 1:**
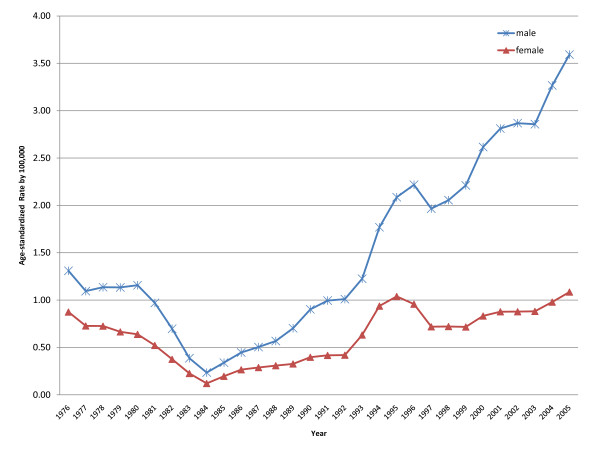
**Trends in age-standardized rates of suicide for males and females (3-year moving averages, centred on the last year in the 3-year period), Philippines, 1974-2005**.

Age-standardized suicide rates for males and females from 1974 to 2005 are shown in Figure 1; male rates were consistently higher than female rates, with the male to female ratio steadily increasing from the mid-80s (from 1.96:1 to 3.31:1 between 1984 and 2005). There was a decrease in the incidence of suicide in the 1970s and early 1980s, but since then rates have been steadily increasing in both sexes; rates rose from 0.23 to 3.59 per 100,000 between 1984 and 2005 in males and from 0.12 to 1.09 per 100,000 in females.

**Figure 2:**
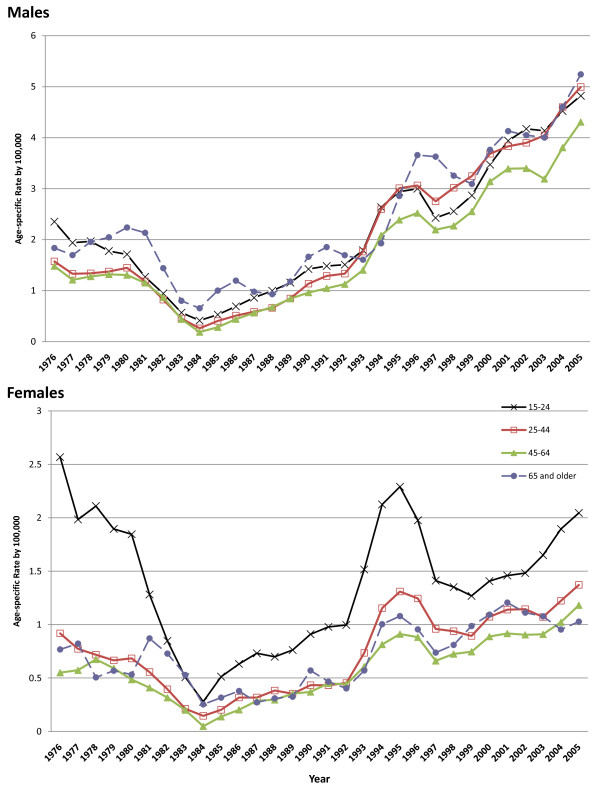
**Trends of age-specific rates of suicide for males and females (3-year moving averages, centred on the last year in the 3-year period), Philippines, 1974-2005**.

Amongst males, rates in all four age groups increased from 1984 onwards (Figure 2); for most of the period rates were highest in men aged 15-24 or 65 and above, but there was little difference between age groups. In females, rates in the 15-24 year olds were 50-100% higher than in the other age groups throughout the study period. The peak in rates in the mid-1990s was seen in all age groups and this was most pronounced in 15-24 year olds.

**Figure 3:**
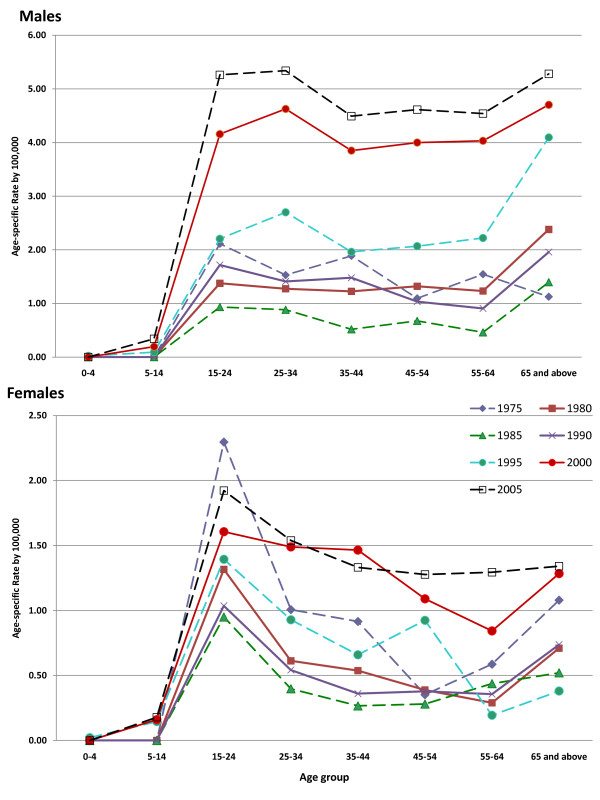
**Age-specific rates of suicide by 5-year intervals, males and females, Philippines, 1975-2005**.

Figure 3 shows how age-specific rates changed in each 5 year period across age groups. For males, a bimodal pattern was observed. In each period the highest rates were generally seen in 15-34 year olds, with a second peak among those aged 65 years and older. Up until 1995, the highest rates among young men were in the 15-24 year olds, but incidence in 25-34 year olds slightly exceeded those in the younger men from 1995 onwards. In females, 15-24 year olds had the highest rates throughout the study period although the difference in rates between 15-24 and 25-34 years olds diminished over time. There was a stepwise decline in rates with increasing age in women, although, as seen in males, rates were somewhat higher in those aged 65 and older than in 45-64 year olds.

**Figure 4:**
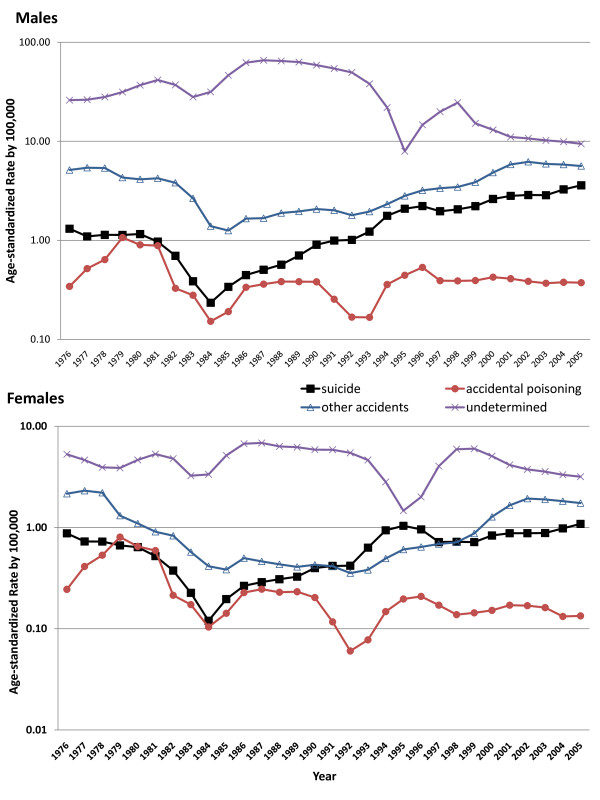
**Trends in suicide, death due to accidental poisoning and other accidents and undetermined injury, males and females, Philippines, 1974-2005**.

Figure 4 shows trends in deaths by cause: suicide, accidental poisoning, other accidents, and deaths of undetermined intent. Rates are plotted on the log scale because of the high incidence of undetermined deaths in the early years of observation. Undetermined deaths were considerably higher than suicides in both sexes. Rates of suicide and other accidents increased from the 1980s onwards in both sexes, whilst deaths of undetermined intent declined - these declines coinciding with the increase in deaths from suicide and other accidents. If, as in other countries, undetermined deaths are mainly suicides, actual suicide rates in 2005 could be as high as 13 per 100,000 for males and 4.3 per 100,000 for females.

In 2005, the ratio of suicide: accidental poisoning: other accidents: undetermined was 1: 0.1: 1.57: 2.62 for males and 1: 0.12: 1.61: 2.92 for females. The decline in undetermined deaths in 1994-1996 corresponded to a small rise in both suicides and other accidents, suggesting some possible misclassification of both suicides and accidents as undetermined deaths.

Although suicide mortality rates by method employed could not be determined from routinely available data, information on the most common methods used were ascertained using published literature and newspaper articles. These were hanging, shooting [17,19] and the ingestion of lethal chemicals, particularly organophosphates [17,18].

### Profile of suicide cases in an indigenous rural area

An anthropological study investigated suicide among the Kulbi people, a tribal group in South Western Philippines (Southern Palawan) based on 18 suicides occurring between 1990-2001 in a population of 867 people - an incidence of 173 per 100,000 per year (95% Confidence Interval, 106-267 per 100,000)[15]. The ratio of male: female suicides was 2:1, the highest numbers of deaths occurred at age 13-29 years for men and 50-70 for women. Most suicides used hanging (65%); the other common methods were poisoning using the juice of *Derris elliptica *(tuba) or an industrial pesticide (tejudan) (21.6%). Among the Kulbi people, reasons for suicide were sickness and old age (among the old), anger, jealousy, or love problems (among younger and middle-aged adults) and grief over death of a loved-one (all ages). While suicide is disapproved of, there is no stigma or consequence attached to it in this community, in contrast to the attitudes and beliefs in the general population.

### Profile of non-fatal self-harm cases

Results from the literature review indicate that 58-77% of cases of non-fatal self-harm presenting to hospitals are female [16-19], the incidence is highest amongst 15-24 year olds, with the peak in females slightly earlier (15-19 years) than in males (20-24 years). The majority of cases are single (53-74%), but 61-84% of these were in a relationship. Half of cases were either students or unemployed (23-29% and 14-26%, respectively).

Between 9-22% of self-harm admissions reported seeing a psychiatrist prior to the attempt. In a study looking at the mental health of people who attempted suicide, 78.7% had adjustment disorders, 7.1% schizophrenia, and 6.2% manic depression [18]. Only 24% and 38% reported intoxication with alcohol or prohibited drugs, respectively, prior to their suicide attempt[16].

Most cases admitted to hospitals (34-56%) had taken overdoses of drugs, such as isoniazid and paracetamol, or ingested chemicals (27-51%) in the form of common household products such as sodium hypochlorite or organophosphates. Self-poisoning was the most common method of self-harm [16-19]. Case fatality among people admitted to hospital following a suicide attempt was 2-3% [16,19].

Family and relationship problems were the most common reasons for self harm, with some of these citing public embarrassments such as being scolded in front of visitors as the main stressor. Around 52-87% of suicide hospital admissions reported having problems with the spouse, boyfriend or girlfriend, or parents.

## Discussion

To the best of our knowledge, this is the first comprehensive summary of the epidemiology of suicidal behaviour in the Philippines. Whilst the incidence of suicide in the Philippines is low compared to other countries [21,22], it appears to have been increasing in recent years, particularly amongst males. Amongst females the highest rates are seen in 15-24 year olds. There is indirect evidence that suicide is under-reported in the Philippines.

The low rates of suicide in the Philippines in the early 1980s could reflect social cohesion during the turbulent Martial Law era and its aftermath [23]. Reductions in suicide rates during periods of war and civil disturbance are well recognised [24]. Recent increases in suicide might be explained by improved reporting and changing social attitudes. Up until 1983 suicides were barred from receiving religious burial rites in the Philippines. The 1983 revision of the Canon Law removed this prohibition [25] and this change coincided with the increase in reported mortality rates. Similar trends and changing attitudes were observed in Ireland which is also predominantly Catholic [26]. Rising trends in suicide in the Philippines are in keeping with the increases seen in a number of Asian countries - most notably Thailand, South Korea, Japan and Hong Kong [22], in contrast many Western countries have experienced reductions in rates since the 1980s [9,27].

The Philippines is a predominantly Roman Catholic country and it is possible that reluctance to report deaths as suicide contribute to the low official rates. Nevertheless, the strong Roman Catholic culture could also contribute to preventing some suicides, due to the beliefs and social norms associated with Catholicism. Similar protective effects of Catholicism have been reported in a recent analysis of data from Switzerland [28]. Likewise, predominantly Catholic countries in Europe such as Portugal, Spain and Italy have amongst the lowest suicide rates in that region.

More women than men attempt suicide in the Philippines, but as seen in most other countries case fatality is higher in males, in part due to males' preference for more violent/lethal methods of suicide. The male-to-female ratio for suicide (3.3:1) in the Philippines is higher than in China or India but comparable to that seen Thailand, Japan and New Zealand [2]. The male: female ratio increased almost two fold between the mid-1980s to 2005. There is no clear explanation for this change, although it could be partly due to persisting poverty and income inequality, combined with increasing labour market competitiveness during this period, despite reported economic growth [29,30]. Other studies have shown that working age men may be more susceptible to suicide in times of economic difficulty than women, possibly due to higher societal pressures to succeed [31].

Suicide attempts and mortality were generally higher in adolescents and young adults than in the older age groups, this contrasts with patterns seen in most countries where rates tend to increase with age [32]. However similar high rates in young people have been reported in Pakistan and Thailand [33,34]. This could be due to increased vulnerability of young people to social stressors [35]. Adolescence is a period of life changes and most teenagers struggle with issues such as independence and developing a sense of identity and a system of values and responsibilities. These struggles are manifest in the high incidence of non-fatal self-harm in this age group worldwide, but in most countries such attempts are generally of lower lethality than attempts made in older age groups, and so are not reflected in statistics for completed suicide. Reasons for this excess in young people in the Philippines require further investigation.

The increasing rates of suicide and accidental deaths and corresponding decrease in undetermined deaths are suggestive of some underreporting and misclassification. More studies, however, are needed to further evaluate possible underreporting. The small peak in the mid-1990s coincided with the passage of the Administrative Order 1, s. 1993, which defined and updates civil registry laws and guidelines [36], this could have resulted in better suicide reporting, if only for a short period of time.

There is a dearth of detailed information on the incidence of suicide and suicide attempts in rural areas but the rate reported in one indigenous population in the 1990s is among the highest in the world (173 per 100,000) [15]. High suicide rates have been reported among indigenous groups in other settings [37]. Although of a larger magnitude, the difference in rates between the Kulbi people and the general population is similar to that observed between the Atayal people and the Taiwanese population [37].

The most commonly used methods of suicide in the Philippines appear to be hanging, shooting and organophosphate poisoning. Although, the lack of method-specific suicide mortality data means that these observations are based on newspaper reports; such reporting is likely to be non-representative and restricted to the most newsworthy and overt suicides. Non-fatal self harm most often involved drugs such as isoniazid and paracetamol, which can be purchased without prescription and in unlimited amounts. Paracetamol is widely used as an analgesic and antipyretic drug, and high use of isoniazid reflects its availability due to the high prevalence of TB in the Philippines [38], where it is known as a vitamin or medication for "weak lungs" (a local euphemism for TB) [39].

The choice of method is greatly influenced by availability. In the Philippines, there is minimal regulation for the sale of over the counter drugs and organophosphate insecticides, although many toxic pesticides are banned in the country [40]. Private possession of firearms is allowed conditional to acquisition of a license, which is not subject to background checks [41,42]; 'home-made' firearms are also in common use[42].

Our study has some limitations, particularly the availability and reliability of routinely reported mortality data. Aside from the reluctance to report suicide deaths due to the stigma, inadequacies have also been noted in death registration practices, and incompleteness and errors in entries for the cause of death in official records[13,43]. Even in medically attended deaths, certification is generally based on the clinical symptoms rather than results of autopsy. Although guidelines for death certificate completion exist, these are often not followed [44].

The profile of people who made suicide attempts was derived from hospital-based studies in selected, mainly urban, areas; patterns may differ in more rural locations. Suicide patterns and beliefs could be different for people in rural areas and for cultural minorities, as seen in the study of the Kulbi people [15]. Furthermore these studies covered different time periods and so the patterns of medicines and poisons taken in suicide attempts may be non-comparable if these have changed over time.

## Conclusions

These findings clearly show the need for more reliable data in order to understand suicide behaviour and establish prevention strategies in the Philippines. Emphasis should also be given on improving cause of death reporting, particularly of suicides. Change in attitudes towards suicide victims should be encouraged to improve reporting. Provision of training for medical students, practitioners and local civil registrars in the need for accurate death certification should also be continued and expanded.

## Competing interests

The authors declare that they have no competing interests. DG is an NIHR Senior Investigator.

## Authors' contributions

MTR conducted data synthesis and analysis and drafted the manuscript, MAL supervised data collection and literature review, DG supervised data synthesis and analysis and the drafting of the manuscript. All authors contributed to the conceptualization of the research, and reviewed and approved the manuscript.

## Pre-publication history

The pre-publication history for this paper can be accessed here:

http://www.biomedcentral.com/1471-2458/11/536/prepub
